# Animal Models in Behçet's Disease

**DOI:** 10.1155/2012/273701

**Published:** 2012-02-27

**Authors:** Ozlem Yildirim

**Affiliations:** Department of Ophthalmology, Faculty of Medicine, Mersin University, 33070 Mersin, Turkey

## Abstract

Behçet's disease is a chronic, recurrent, multisystemic, inflammatory disorder affecting mainly the oral and urogenital mucosa and the uveal tract. Although the etiology and pathogenesis of Behçet's disease are unknown, numerous etiologies have been proposed, including environmental, infectious, and immunological factors; an autoimmune basis, characterized by circulating immune complexes and complement activation, has gained increasing acceptance. To test and understand immunopathogenesis of Behçet's disease, animal models were developed based on enviromental pollutants, bacterial and human heat shock protein derived peptides, and virus injections. Using these animal models separately and/or concurrently allows for a more effective investigation into Behçet's disease. Animal models developed in the last 10 years aim at the development of efficient and safe treatment options.

## 1. Introduction

Behçet's disease (BD) is a chronic, multisystemic, inflammatory disorder and is characterized by mucocutaneous, ocular, arthritic, vascular, gastrointestinal, and central nervous system involvement. The disease has a chronic course with periodic exacerbations and progressive deterioration [1].

Since the dermatologist Dr. Hulusi Behçet [2] comprehensively described this disease involving multisystemic organs in 1937, the etiology of BD has still remained unclear. Various hypotheses have been proposed centering on viral infection, autoimmune disease, streptococcal-related antigens, specific alleles of the human major histocompatibility complex, genetic factors, and hazardous chemicals [3–7]. The history and recent developments in the immunopathogenesis of BD are reviewed and discussed in this paper.

## 2. Short History

Viral infection has long been postulated as one of the etiologic and triggering factors. Hulusi Behçet proposed that the disease was caused by a special virus. Although he was unable to demonstrate one, he had observed intracellular inclusion-like forms in smears from the hypopyon of the anterior chamber and aphthae [2]. In 1953, Sezer [8] was the first to isolate the virus from ocular fluid and serially cultivate it in chorioallantoic membrane of fertile eggs. He inoculated the material from the ocular fluid of patients into brains of mice. Inoculated mice showed manifestation such as roughening of the coat, inactivity or hyperactivity, tremor, circling, paralysis, encephalitis, thrombophlebitis, and swelling. Evans et al. [9] isolated the virus from the eye and brain of a patient who died of the disease. Mortada and Imam [10] found inclusion bodies from scrapings of the scrotal and buccal ulcers, as well as from the hypopyon fluid. When the fluid from the scrapings of scrotal and buccal ulcers and hypopyon was inoculated into the chorioallantoic membrane of 10-day-old chick embryos and incubated for 2 days, whitish plaques were seen. These plaques showed inclusion bodies exactly like those seen in scrapings. The filtrates from plaques were inoculated intracerebrally into 3-week-old white Swiss mice. Seventeen out of 21 mice died while 5 control mice inoculated with saline remaining alive.

Eglin et al. [11] using method of in situ hybridization, detected RNA complementary to herpes simplex virus (HSV) type 1 in the mononuclear cells of patients with BD. HSV type 1 DNA was detected in the whole blood by Bonass et al. [12] with dot blotting technique. Denman and colleagues [13] detected HSV DNA with southern hybridization using Eco R1 digested DNA from the peripheral blood mononuclear cells of patients with BD. In 1991, Studd et al. [14] detected HSV-1 DNA by polymerase chain reaction (PCR) in peripheral blood leukocytes of BD patients with recurrent oral ulcers.

Lee et al. [15] tried to detect HSV DNA in saliva of patients with BD, and to evaluate whether the presence of HSV in saliva is associated with the presence of an intraoral ulcer, and to investigate any possible relationships between HSV and BD using PCR. The results from saliva showed that almost 40% patients were positive for HSV DNA, compared to 14% of healthy controls.

Lee and colleagues [16] investigated the relationship of intestinal ulceration of BD and HSV. PCR results were all positive in specimens of patients with BD. Bang et al. [17] experimented to detect HSV DNA from ulcerative genital tissue of patients with BD. They applied 8 cases and all showed HSV bands. From these reports, we can conclude that there is a relationship between HSV DNA and ulceration of various epithelial tissue of BD.

## 3. Animal Models Aiming to Elucidate the Immunopathogenesis of Behçet's Disease

Animal models are very important and necessary in most fields of research. Many investigators have tried to develop an animal model for use in BD. In 1979, Hori and colleagues [4] created typical BD-like lesions in Pitman-Moor's strain, miniature swine treated with agricultural chemicals such as organophosphate, organochloride, and inorganic copper. The total number of animals used for the development of an animal model by Hori and colleagues was 8. Though all of the experimental animals showed BD-like symptoms after a 1-year administration of the chemicals, it is not feasible to breed such a large group for use in other experimental design.

**Figure 1:**
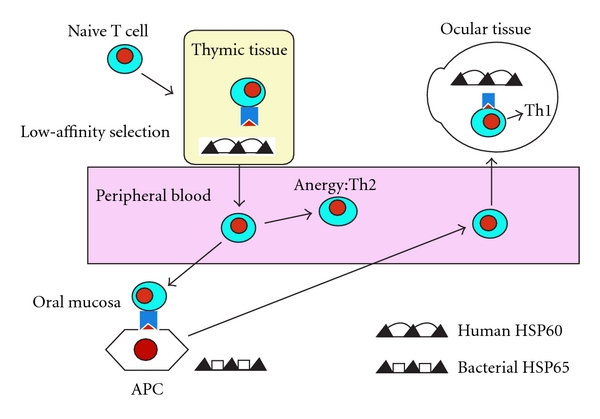
Role of bacterial and human heat shock protein (HSP) 60/65 as T cell antigens in Behçet's disease. APC: antigen presenting cells.

Heat shock protein (hsp) has also been implicated in the pathogenesis of several human and experimentally induced autoimmune diseases such as BD, both as target antigens and as intracellular chaperones involved in peptide binding to HLA antigens [18] (Figure 1). Lehner et al. [19] reported that uveitis could be induced in Lewis rats by 4 peptides derived from the sequence of the mycobacterial 65 kD hsp, which stimulate specifically TCR *γδ* lymphocyte from patients with BD and human 65 kD hsp derived peptides. Lehner groups' Lewis rats showed only single eye symptom. Patients' symptoms of BD are multiple, chronic, or recurrent. If additional symptoms were to appear in hsp derived peptide stimulated Lewis rats, this animal model would be more useful. 

Before using the ICR mouse strain, Sohn et al. tried guinea pig and Sprague-Dawley rat models. They used subcutaneous injections in back skin and the scrotum, intramuscular injections in the tongue, and a direct swab on the eyeball. Using these methods, no symptoms similar to BD appeared. They thought that the key point for success was the correct identification of the route of infection of HSV. The route of HSV infection is an important factor affecting multisystemic expressions of the symptoms.

In 1998, Sohn et al. [20] experimented to develop an animal model for use in BD using HSV infection which was hypothesized to be one of the etiologic or triggering factors in BD by Lee et al. [15, 16]. To test the HSV infection hypothesis and to develop an animal model, ICR mice were inoculated with HSV. Using method of Hirata and colleagues [21], the earlobes of 258 mice of ICR strain, aged 5 to 6 weeks, were scratched with a needle, then inoculated with 1.0 × 10^6^ plague forming units (pfu)/mL of HSV type 1 (KOS strain) solution. As a control, 30 mice were inoculated in the same site with a culture medium. Four weeks later, a second inoculation was performed using the same methods, followed by 16 weeks of observation. The mice inoculated with HSV manifested changes 3 to 4 days after the first inoculation. The signs that appeared most rapidly and commonly were partial hair loss in the face region and erythema on the scratched earlobe. Other common symptoms were skin ulceration of the earlobes, scruff, and genitalia and eye symptoms including keratitis, conjunctivitis, and uveitis. Mice with relatively severe symptoms showed poor general condition and soon died. Some mice did not show any visible symptoms, but appeared to be in poor general condition, they also died. After the second inoculation, arthritis, oral and genital ulcers, and keratitis were the main symptoms. These symptoms were generally more severe than those appearing after the initial inoculation. Two or more symptoms in one mouse were considered an indication of a BD-like syndrome. After the induced infection, 86 mice (33.3%) died, 77 (29.8%) showed BD-like symptoms, and 95 (36.8%) had a healthy normal appearance or a single symptom. The symptoms included skin ulcers on the earlobe, scruff, abdomen, back, or face (57.1%); eye symptoms (39.0%); partial hair loss (33.8%); genital ulcer (19.5%); bullae (11.7%); arthritis (5.2%); gastrointestinal ulcer (5.2%); tongue ulcer (3.9%). The induced BD-like symptoms were similar to the clinical manifestations of ulcers, uveitis, and arthritis which have been significant in diagnosing BD in patients. Aside from hair loss, the most frequent symptoms affecting mice were skin ulcers, eye symptoms, and genital ulcers. The frequency of these symptoms were similar to that of patients with BD.

The PCR was used to detect HSV DNA sequences in DNA extracted from the lesions of mice with BD-like symptoms. The methods were almost the same as those used in the patient experiment. HSV DNA sequences were detected in the lesional skin and gastrointestinal track, but not in normal healthy skin area. Abdominal skin lesions stained with hematoxylin and eosin showed that many inflammatory cells had accumulated around the blood vessel. Vasculitis was also common in intestinal, oral, earlobular, and genital epithelial lesions. These findings were very similar to typical morphological changes in human BD [22]. These experiments proved that it was possible to induce BD-like symptoms such as ulceration and vascular inflammation in ICR mice by inoculating them with HSV. For this reason, many researchers have used this animal model to understand the etiopathogenesis of BD and efficiency and safety of newly developed drugs in BD.

In 1978, Ohno and Sugiura [23] reported an association between human leukocyte antigen (HLA) and BD in Japan. Then, several authors have presented evidence of an HLA association with, and HLA B51, one of the split antigens of HLA B5, was found to be the most strongly associated genetic marker [24–26]. To pursue the correlation between viral infection and genetic factors in the development of the disease, several inbred mouse strains—B10.BR, B10.RIII, C57BL/6, C3H/He, and Balb/c—which had different haplotypes of major histocompatibility complex (MHC), were inoculated with HSV type 1 (KOS strain) using the method of Sohn and coworkers [27]. BD-like symptoms appeared in B10.BR, B10.RIII, and C57BL/6 as in the case of ICR mouse strain previously reported by authors as a BD mouse model [20]. These mouse strains manifested single or multiple symptoms. Manifestations of oral, genital, skin ulcers, uveitis, and arthritis, that are clinically significant for the diagnosis of BD in patients appeared. The lesions spontaneously healed and recurred repeatedly. According to the revised Japanese criteria [28], the manifestations in mice were classified into major and minor symptoms. Among these symptoms, having more than two symptoms (including and above one major and one minor symptom) were diagnosed as BD-like symptoms. The mice with a single symptom were classified as the normal group. More than 40% of B10.BR, B10.RIII, and C57BL/6 showed BD-like symptoms, compared to 2% of C3H/He and Balb/c. Inbred mice with different haplotype of the H-2 region showed similar incidence rates of BD-like symptoms except C3H/He and Balb/c strains. The results from these inbred mouse strains in induction of BD-like symptoms are more important than MHC association.

Although viral infection has long been postulated as a contributing factor in the etiology of BD, and viral involvement has been demonstrated, viral infection alone is not sufficient to explain the pathogenesis of BD. Some evidence suggests that immunologic abnormalities are also important. Sohn and coworkers [29] attempted to determine whether inactivation of macrophages influences the development of BD and whether related cytokines play a role in the modulation of BD symptoms. As previously described, an animal model was developed by using ICR mice [20]. HSV-1 inoculation was done twice 10 days apart, which was followed by 16 weeks of observation. Mice with ≥1 major and 1 minor symptom were classified as having BD. Liposome-encapsulated clodronate (lip-Cl_2_MDP) was injected intravenously in mice to inactivate their macrophages. Animals treated with HSV combined with lip-Cl_2_-MDP had a lower incidence of BD-like symptoms than did those treated with HSV alone. These results suggest that macrophages may play an important role in the development of this disease. Macrophage deletion did not seem to affect mortality in 2 groups. The suppression of the development of BD-like symptoms was correlated with the induction of interleukin (IL-) 4 expression in mouse. When the Th2 adjuvant ovalbumin-(OVA-) alum was injected into mice with BD-like symptoms, their cutaneous symptoms improved. Adoptive transfer with splenocytes from OVA-alum injected mice also resulted in improvement. These findings suggest that up-regulated Th2 cytokine expression induced by macrophage inactivation may be closely related to the development, deterioration, and improvement of BD induced by HSV.

Neutrophil activation is one of the immunopathogenesis aspects of BD. Neutrophils have a pivotal role in innate immune responses. As typical BD lesions such as pustular folliculitis, pathergy reactions, and hypopyon have significant neutrophil infiltrations, neutrophil functions and activation status have been investigated [30]. There are conflicting reports of increased, normal, or decreased basal and fMLP stimulated superoxide productions, phagocytosis, chemotaxis, and neutrophil-endothelial adhesion in BD. In HLA-transgenic mic presumed model for BD, the only abnormality seen is increased superoxide release in response to fMLP. High superoxide responses were also present in HLA-B51+ patients and healthy controls in the same study.

In the etiology and pathogenesis of BD, immunological factors, an autoimmune basis, characterized by circulating immune complexes and complement activation, has gained increasing acceptance and significance. Previous work demonstrated the presence of antibodies to guinea-pig oral mucosal cells by immunofluorescence [31]. Another work has demonstrated oligoclonal T-cell expansion in patients with BD, suggesting an antigen-driven immune response [32]. Immunisation with retinal S antigen or interphotoreceptor retinoid binding protein (IRBP) causes an experimental autoimmune uveoretinitis that resembles some human uveitic conditions [33, 34]. In vivo or in vitro sensitised, S antigen or IRBP specific T cells transferred to naive animals induce experimental autoimmune uveoretinitis [35]. On the other hand, Yamamoto and coworkers found that patients with BD with uveitis exhibited the highest and the most frequent positive responses to S antigen and IRBP, as well as to peptide M, a main uveitogenic site of S antigen [36]. Then, another study showed that patients with BD without uveitis did not differ in their responses to S antigen from the responses in the control group [37]. Yet, more responders to IRBP were observed in the patients group without uveitis (35%) than in the control group (14%), although their responses were lower than the responses observed in patients with uveitis. The presence of lymphocyte responses to retinal antigens in patients with BD without uveitis might indicate a preclinical stage of ocular involvement. Thus, these data support the idea that autoimmunity to retinal specific antigens may play a role in the ocular inflammation in BD.

Immunological responses to four T-cell and B-cell epitopes have been identified within the mycobacterial 65-kDa hsp in patients BD [38]. The four mycobacterial T-cell epitopes show significant homology with the human 60-kDa mitochondrial hsp [39]. Some groups have found T-cell proliferative responses to human hsp60 and peptides derived from it [40], others have found antibodies to Yersinia derived hsp60 [41]. Lehner [42] have characterized gamma-delta T-cells reactive against several peptides of human hsp60 in patients with BD. In 1994, Stanford et al. [43] investigated mycobacterial and homologous human hsp T-cell peptide epitopes specific for T lymphocytes in BD for their pathogenicity in Lewis rat. The potential pathogenicity of eight peptides and two controls was assessed by administering the peptides in enriched Freund's adjuvant into the footpads of male Lewis rats. In this study, they have shown that ocular signs may be induced in Lewis rats by injecting synthetic peptides derived from the sequences of the mycobacterium tuberculosis 65-kd hsp, and with greater frequency by using the homologous human hsp peptides. Mild or moderate clinical disease in these animals appears to be more common in the anterior segment of the eye. But, clinical involvement of the mouth, skin, external genitalia, joints, or the neurological system was not observed. The peptides that are most frequently recognized by T lymphocytes from patients with ocular type of BD (136–150 and 336–351) produced the highest incidence of disease in the experimental rats. Then, in 1998, Hu et al. [44] showed that human 60-kDa hsp-derived peptide 336–351, which is specific in stimulating T cell responses in BD, induced uveitis in Lewis rats when administered orally and nasally. The mucosal route of induction of uveitis is more likely to mimic the clinical situation, in which hsp of oral microorganisms, such as streptococci, may elicit immune responses which cross-react with mucosal hsp and initiate pathological changes [38]. Besides, the experiments to prevent the development of uveitis by oral or nasal administration of peptide have failed. In addition, administration of monoclonal antibody against CD8 or CD4 revealed that, whereas monoclonal antibody against CD8 enhanced uveitis, against CD4 suppressed uveitis. Thus, CD4 cells mediate whereas CD8 cells suppress the development of uveitis.

Based on these studies, Mor and colleagues [45] initiated a study to seek target antigens associated with the tissues involved that might be pathogenic in laboratory animals. They tested patient sera for the presence of antibodies to antigens found in lysates various tissues. They identified a subset of patients with immune reactivity to a 37-kDa antigen present in the skin, tongue, vagina, muscle, and heart rat tissue. In-gel digestion and mass spectrometry revealed the antigen to be *α*-tropomyosin. To test whether induction of autoimmunity to a *α*-tropomyosin might be pathogenic, Lewis rats were immunized with bovine *α*-tropomyosin in complete Freund's adjuvant (CFA). The immunized rats developed lesions in the uveal tract and skin, with features of BD. Control rats injected with PBS/CFA emulsion did not develop uveitis or skin inflammation.

## 4. Animal Models Designed to Investigate the Efficiency of Newly Developed Drugs

Animal models have been used for evaluating the efficiency of newly developed drugs as well as investigating the disease's etiopathogenesis. The BD-like mouse model, developed by Sohn et al. has been found to show immunological abnormalities [27, 29], and thus is a valuable tool to study the effect of various therapeutic drugs. Treatment with the antiviral agent aciclovir has failed to alleviate the frequency and severity of orogenital ulceration and or other disease features in BD [46]. Sohn and coworkers [47] administered famciclovir, an antiviral compound that acts against HSV, varicella-zoster virus, and hepatitis B virus, in the HSV-induced BD mouse model, to demonstrate the efficiency of famciclovir. Using the HSV-induced BD mouse model [20], famciclovir was administered variously before and after inoculation of from the day of lesion occurrence, with appropriate controls. Ulceration of the mouth and genital skin and eye involvement were monitored. In addition, spleen cytokine expression was measured by PCR. Pretreatment or concurrent treatment with famciclovir did not attenuate the occurrence of BD symptoms. However, administration of famciclovir from the day of lesion occurrence was effective in about 60% of mice with single symptom and 40% of those with BD symptoms and preventing recurrence. But recurrence rate was higher in BD mice. Oral and genital ulcers did not recur, contrasting with skin ulceration and eye involvement which recurred despite administration of famciclovir. Therefore, the overall rate of improvement in BD was lower than the rate in cases showing only a single symptom. To determine whether or not the improvement of symptoms by famciclovir correlated with the expression of cytokines, RT-PCR was performed on the spleens of improved and relapsed mice after administration of famciclovir. IL-2 was expressed in relapsed mice that had areas of ulcerated skin, while it was not expressed in the improved mice. Interferon-(IFN-) *γ* was always expressed and was not related to the improvement of ulceration. IL-4 and IL-10 were not expressed at any time following administration of famciclovir. These findings suggest that famciclovir might be a candidate for controlled clinical trials in the human form of BD.

In another study, thalidomide was administered in order to understand the mechanism for the improvement in symptoms in BD-like mice [48]. Despite its inherent teratogenic risk, thalidomide has proven to be of clinical use in a small number of immunological disease, including BD [49]. However, the mechanism of action of thalidomide in patients with BD remains poorly understood. In this study, a BD-like mouse produced by HSV inoculation, previously published by Sohn et al. [20] was used. ICR mice were inoculated twice with HSV over a 10-day period. Thalidomide (100 *μ*g) was orally administered to ten BD-like mice for five consecutive days. Placebo was administered to ten BD-like mice in an identical manner. Eight out of ten thalidomide-treated mice showed improvement in skin ulceration, bullae and crusting, and intestinal and genital symptoms. The control group, treated with PBS instead of thalidomide, did not show any change in their BD-like symptoms. The mice were sacrificed on the 6th day, and the spleens were subjected to RT-PCR, FACS, western blot, and immunohistochemical analysis. Tumor necrosis factor-(TNF-) *α*, macrophage inflammatory protein-(MIP-) 1*α*, perforin, and Fas were influenced by thalidomide treatment. These results suggest that thalidomide can attenuate HSV-induced BD-like symptoms in mice through the downregulation of TNF-*α* (*P* < 0.005) and the upregulation of MIP-1*α* (*P* < 0.005), perforin (*P* < 0.05), and Fas receptor (*P* < 0.1).

Beneficial therapeutic effects have been reported for colchicine, thalidomide, cyclosporin A, IFN-*γ*, and systemic corticosteroids in the treatment of BD. The nucleoside analog gemcitabine (2′,2′-difluorodeoxycytidine, dFdC) is a new significant immunosuppressive agent that may be useful not only in graft-versus-host disease but also in autoimmune diseases. dFdC is a nucleoside analog affecting the pyrimidine pathway. Sohn et al. designed the study in order to examine the effects and side effects of gemcitabine on skin lesions of HSV-induced BD-like mouse model [50]. For the dose-escalation study, healthy ICR mice were treated intraperitoneally with dFdC over 5 consecutive days. For the efficacy study, ICR mice were inoculated with HSV as described earlier and classified as having BD according to a revised Japanese classification [20], and then 18 BD mice were randomly assigned to placebo, 0.06, or 0.12 *μ*g of dFdC/day over 5 days, applied intraperitoneally. Serum levels of IL-4, IL-6, IL-10, IFN-*γ*, and TNF-*α* were determined using enzyme-linked immunosorbent assay. After application of 3 *μ*g of dFdC over 5 days, alanine aminotransferase increased (*P* = 0.032), but all other kidney and liver parameters were unchanged. In BD-like mice, soon after 5 days of dFdC treatment, cutaneous manifestations ameliorated by more than 60% (*P* = 0.017), depending on the dFdC dose applied but not in the control mice. There was no significant change in cytokine levels after 5 days of dFdC treatment compared to pretreatment levels but only a trend toward reduced IL-10 under dFdC treatment (*P* = 0.135), and none of the cytokine levels correlated with response to treatment. Moreover, dFdC shows promising effects to improve cutaneous lesions in the HSV-induced BD-like mouse model. In this animal model, effects of dFdC on cytokine profile remained inconclusive.

In recent years, other options of the treatment of BD are biologic agents. TNF-*α* is a potent paracrine and endocrine mediator of inflammatory and immune functions. TNF-*α* overexpression has been implicated in acute and chronic inflammatory diseases, such as septic shock, bowel disease, Crohn's disease, rheumatoid arthritis, atopic dermatitis, psoriasis, and BD [51]. TNF-*α* is produced primarily in T cells, polymorphonuclear cells (PMNs), dendritic cells, and macrophages [52]. In macrophages, TNF-*α* gene expression is induced by physical, chemical, and biologic stimuli that include ischemia, trauma, irradiation, viruses, bacteria, tumor cells, complement, and cytokines. Rapidly supplanting antisense methods [53, 54]. RNA interference (RNAi) is a recently discovered process that utilizes either endogenous or exogenous, double-stranded RNAs to inhibit expression of genes in a highly sequence specific manner. In mammals, RNAi can be invoked by introducing short (19–21 nucleotide), double-stranded RNA oligonucleotides, referred to as small interfering (siRNAs), or silencing RNA molecules, of a sequence complementary to that of the target gene. The siRNAs are bound by an RNA inducing silencing complex in the cytoplasm and silence the expression of the target mRNA. Therefore, RNAi offers promise as a novel therapeutic device and in addition may be used as a tool in functional genomics studies to elucidate genes controlling disease pathways [55]. To inhibit the expression of TNF-*α*, Choi et al. [56] used siRNAs to reduce over expression of TNF-*α* in vitro in cell cultures and in vivo BD-like mouse model for amelioration of chronic inflammation. Male ICR mice were infected with HSV-1, as previously described [20]. TNF-*α* siRNA was injected intraperitoneally twice with 1-week interval. To compare the efficacy of TNF-*α* siRNA versus an anti-TNF-*α* antibody, Infliximab, and TNF-*α* receptor, etanercept were administered to symptomatic mice with inflamed tissue, which were subsequently observed for 2 weeks. Infliximab, at 150 *μ*g/mouse (5 mg/kg), was intravenously injected only once. Etanercept (25 *μ*g/mouse, 50 mg/60 kg) was injected subcutaneously twice per week. Other BD mice were treated with scrambled siRNA or were untreated as negative controls. Intraperitoneal delivery of TNF-*α* siRNA effectively decreased BD symptoms in 18 of 32 cases (56.3%). Scrambled siRNA treatment decreased BD symptoms in 2 of 19 cases (10.5%). Infliximab was effective in 11 of 27 cases (40.7%) and Etanercept was also effective in 9 of 25 cases (36.0%) at the end of the second week after treatment. TNF-*α* siRNA reduced serum levels of TNF-*α* (1.57 ± 0.43 pg/mL), compared to levels in mice not injected (84.02 ± 24.59 pg/mL) (*P* < 0.01) or scramble injected (118.89 ± 20.08 pg/mL) (*P* < 0.01). A significant reduction in TNF-*α* level was observed as early as 24 h after treatment, and the level did not recover until 2 weeks after treatment, thus demonstrating an immediate, potent, and lasting biologic effect of siRNA treatment. After single injection of TNF-*α* siRNA, improvement of BD symptoms showed at 9 ± 7th day on average, contrary, in infliximab-injected group, improvement was apparent at 15 ± 4th day after injection (*P* < 0.05). Choi et al. [56] show that siRNA can be employed to inhibit cytokine gene expression in an in vivo disease mouse model. This inhibition may, therefore, be attributed to the improvement of inflammatory symptoms.

TNF-*α* plays a central role in a variety of inflammatory responses [57]. TNF-*α* and adhesion molecules have been shown to be upregulated in BD patients [58, 59]. In addition, downregulation of TNF-*α* using anti- TNF-*α* antibody (infliximab) was found to suppress various BD symptoms including sight-threatening panuveitis [60] and intestinal ulcers in patients [61] and mice [62]. Another TNF-*α* blocker, etanercept, is also known to be an effective therapeutic agent for BD patients [62] and mice [56]. To test for possible anti- TNF-*α* and anti-inflammatory activities, Choi et al. [63] administered synthesized pyridine compound derivatives (SK94, SK126) from a natural lead source to mice. Lipopolysaccharide-(LPS-) induced TNF-*α* production was analyzed in the endothelial cells, Raw 264.7 cells, and serum of normal mice after treatment with SK compounds. ICR mice were inoculated with HSV as described earlier and classified as having BD according to a revised Japanese classification [20]. SK compounds (1 mg) or thalidomide (50 *μ*g) were orally administered to five to ten BD-like mice for five consecutive days. Placebo was administered to ten BD mice in an identical manner. The results of the present study that pyridine compound derivatives can downregulate the expression of TNF-*α* and adhesion molecules in normal mice. Furthermore, these compound downregulated TNF-*α* and adhesion molecules in BD mice and ameliorated the symptoms. Orally administered SK compounds were found to be abel to effectively downregulate the serum level of TNF-*α* in LPS treated Balb/c mice and BD mice. Additionaly, SK126 more efficiently downregulated the serum level of TNF-*α*, soluble intercellular adhesion molecule-1 (sICAM-1), and soluble E-selectin when compared to thalidomide in BD mice. SK126 and SK94 more efficiently downregulated sICAM-1, soluble vascular cell adhesion molecule-1 (sVCAM-1), and soluble E-selectin when compared to thalidomide in the sera of BD mice and it downregulated soluble E-selectin when compared to the thalidomide treated group in the spleen tissues of BD mice. SK126 and SK94 decreased monocyte chemotactic protein-1 (MCP-1) in the spleen tissues of BD mice. The change in the severity score of SK94-treated BD mice was similar to that of the thalidomide treated-group. The decreasing inclination of severity score was steeper in the SK126-treated group than the thalidomide-treated group, which demonstrates that SK126 more effectively improves BD symptoms than thalidomide. Overall, SK126 is more effective and safer than thalidomide and more convenient than infliximab or etanercept as a treatment for BD. The downregulated cell adhesion molecules and TNF-*α* were correlated with the amelioration of BD symptoms in mice. These findings suggest that SK compounds can be used as therapeutic agents to reduce the levels of TNF-*α* and adhesion molecules during the treatment of inflammatory disease, especially through oral administration.

In conclusion, although many human and animal studies have been made to clarify etiology and pathogenesis of BD since the disease was first described in 1937, the disease's etiopathogenesis is still controversial. Animal models developed so far has been insufficient to describe both the clinical aspects and etiopathogenesis of the disease. An ideal animal model has not been developed yet. Therefore patient reports issued in the literature related to BD provide more valuable information compared to experimental studies. Besides these animal models may helped to determine the disease's efficient and safe treatment alternatives. Better characterisation of pathogenic immune cell subsets, systematic and local antigens, and abnormal cell-activation mechanisms may help in the future to develop more specific and less toxic immunotherapeutic approaches to the still unsatisfactory treated BD.
